# Epidemiological features of 1,332 cases of hip fracture in Shanghai, China (2015–2020)

**DOI:** 10.1186/s42836-024-00236-4

**Published:** 2024-04-01

**Authors:** Miaomiao Yang, Ying Zhang

**Affiliations:** Department of Nursing, Zhongshan Hospital Fudan University, 180 Fenglin Road, Xuhui District, Shanghai, 200032 China

**Keywords:** Hip fracture, Retrospective analysis, Epidemiology

## Abstract

**Purpose:**

This study aimed to analyze the epidemiological characteristics of hip fracture in all age groups in Shanghai, and to evaluate the hospitalization cost of patients with hip fracture.

**Methods:**

A total of 1,332 hip fracture patients admitted to a tertiary general hospital between January 2015 and May 2020 in Shanghai were included. Age, sex, diagnosis, cause of injury and site, fracture type, comorbidities, length of stay, treatment, outcomes (at discharge) and hospitalization expenses were recorded. The epidemiological characteristics of hip fracture were analyzed by using SPSS 26.0 software.

**Results:**

The average age of hip fracture was 77.24 ± 12.66 years, and 69.0% of the patients were female. Overall, 886 patients had femoral neck fracture, and 446 patients suffered from intertrochanteric fracture. Most of the fractures caused by falls at the same level and falls from a height occurred in those aged 81–90 years; and traffic accident injuries mostly took place in patients aged 50–60. Among the 1,302 hip fracture patients who underwent surgical treatment, hip replacement was the major choice for femoral neck fracture, accounting for 49.2%. Internal fixation was the main treatment choice for intertrochanteric fracture, making up 97.8%. The median length of hospital stay lasted 8 days and at cost of hospitalization was ¥49,138.18 RMB.

**Conclusion:**

This epidemiological study found that patients with hip fracture had certain distribution characteristics in age, sex, type of fracture, injury season, cause of injury, mode of operation, length of stay, cost, and so on. Proper medical management, social preventive measures, and prevention of falls are needed to reduce the risk of hip fracture and the socioeconomic burden.

## Introduction

Hip fracture refers to a fracture of the proximal femur, including femoral neck fracture and intertrochanteric hip fracture. With the population aging, hip fracture has been incrementally becoming a public health issue [1]. Its high morbidity and mortality pose not only a threat to the life and quality of life of the elderly population [2, 3], but also a huge economic burden on the society at large [4, 5]. Studies have found that the mortality rate at 1-year after hip fracture was up to 22% [6]. Among the survivors, 50% lost their functional independence, and one-third eventually became fully dependent [7].

Globally, a great many national and institutional studies have epidemiologically assessed hip fracture [8–10] but only few reported its epidemiology and socioeconomic costs in China. Of all those studies, some had a limitation of small sample (830 patients [11], 938 [12] patients), and some covered only a short period of time (1 years [13], and 4 [14] years). Only one study reported the epidemiology at the national level, but it only included patients aged ≥ 65 years.

It is estimated that, across the globe, the case number of hip fractures will rise to 4.5 million by 2050, about half of which are likely to occur in Asia, and especially in China [15]. Shanghai is one of the populous cities in China, and has a large aging population. The purposes of this study were to analyze the epidemiological characteristics of hip fracture among all age groups in Shanghai, and to evaluate the hospitalization cost of hip fracture patients.

## Methods

### Study design

This retrospective observational cohort study analyzed the medical records of all hip fracture patients from a tertiary general hospital between January 2015 and May 2020 in Shanghai. Patients with periprosthetic and/or pathologic fractures were excluded, and only those who had a history of hip trauma, and were diagnosed with femoral neck fracture (ICD-10, S72.0) or intertrochanteric fracture (ICD-10, S72.1), by X-ray or CT, were included. The following data were recorded: age, sex, diagnosis, cause of injury and site, fracture type, length of stay, treating methods, and hospitalization expenses. Study data were taken from the electronic medical records using hospital computer software. Informed consent was waived owning to the observational nature of this study.

### Statistical analysis

Data are presented as means ± SD for continuous variables with a normal distribution, as median (interquartile range) for continuous variables with a non-normal distribution, and as frequency (%) for categorical variables. The Kolmogorov-Smirnov test was used to assess normal distribution. Student’s t-test and the Mann-Whitney U-test were employed to compare continuous variables with normal and non-normal distributions, respectively. The Chi-square test or Fisher’s exact test was conducted to compare categorical variables.

## Results

### Demographic and clinical characteristics of the participants

**Fig. 1:**
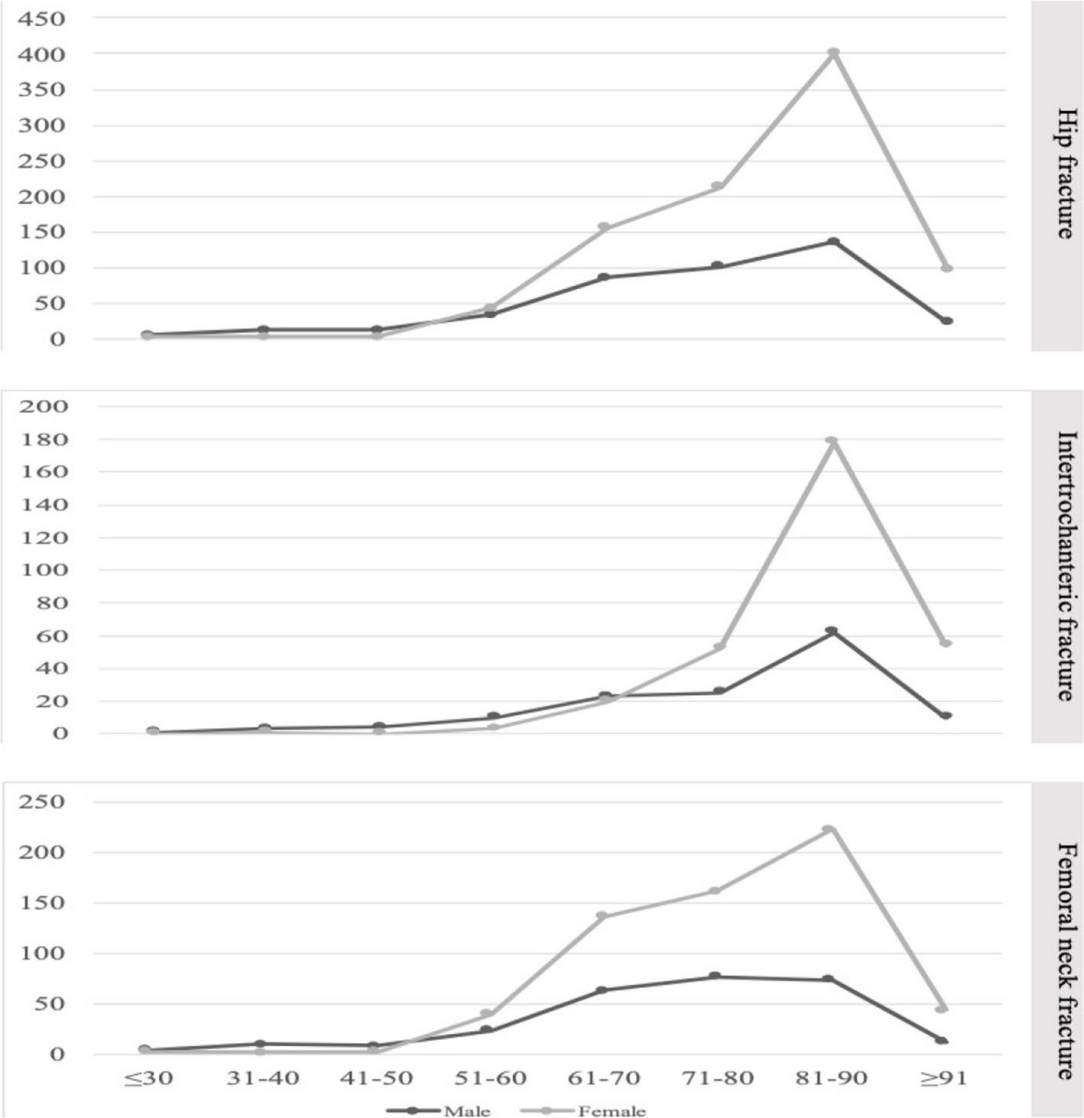
Gender distribution of patients with different fracture types in different age groups

One thousand three hundred thirty-two patients were included in this study. The average age of hip fracture was 77.24 ± 12.66 years, and 69.0% of the patients were female. Overall, 886 patients had femoral neck fracture, and 446 patients suffered from intertrochanteric fracture. The median age of intertrochanteric fracture group was 84 years, which was significantly higher than that of femoral neck fracture group (*P* < 0.001) (Table 1). Among different fracture types, the gender distribution of different age groups showed statistically significant differences (*P* < 0.001). When the age was over 50 years old, the female patients with femoral neck fracture outnumbered their male counterparts, and when the age was over 70 years old, women with intertrochanteric fracture were more than men with the fracture (*P* < 0.001, Fig. 1).

**Table 1:** Overview of patients and injury epidemiology in relation to fracture type

	Femoral neck fracture (*n* = 886)	Intertrochanteric fracture (*n* = 446)	χ^2^/Mannwhitney-U	*P* value
Gender			0.001	0.971
Male	31.0% (275/886)	30.9% (138/446)		
Female	69.0% (611/886)	69.1% (308/446)		
Age (years) M (P_25_, P_75_)	77.0(68.0, 85.0)	84.0 (78.0, 88.0)	-9.864	<0.001
Injury mechanism			14.770	0.002
Fall at same level	89.1% (789/886)	93.3% (416/446)		
Fall from height	1.7% (15/886)	2.9% (13/446)		
Traffic accident	1.6% (14/886)	1.4% (18/446)		
Other	7.7% (68/886)	2.9% (13/446)		
Season			5.084	0.166
Spring	26.9% (238/886)	29.1% (130/446)		
Summer	20.4% (181/886)	17.9% (80/446)		
Autumn	23.8% (211/886)	20.0% (89/446)		
Winter	28.9% (256/886)	33.0% (147/446)		
Perioperative blood transfusion			171.49	<0.001
No	76.2% (765/1004)	23.8% (239/1004)		
Yes	36.9% (121/328)	63.1% (207/328)		
Treatment type			648.177	<0.001
non-surgical treatment	2.5% (22/886)	1.8% (8/446)		
Internal fixation	24.4% (216/886)	97.8% (436/446)		
Hemiarthroplasty	23.9% (212/886)	0.2% (1/446)		
Total hip replacement	49.2% (436/886)	0.2% (1/446)		
Length of stay(days)	8.0 (6.0,11.0)	7.0 (5.0,10.0)	2.229	0.342
Hospitalization expenses (¥)	58748.1 (39042.8, 73558.3)	42589.5 (37242.7, 48206.8)	-11.283	<0.001

### Distribution of causes of injury

The causes of injury were divided into fall at the same level, fall from a height, traffic accident, and others, with causes of injury categorized by age group shown in Table 1. There were significant differences in age distribution among different causes of injury (χ^2^ = 14.770, *P* = 0.002). The majority of the fractures caused by fall at the same level and fall from a height occurred in subjects aged 81–90 years; and traffic accident injuries mostly took place in those aged 50–60.

### Season of fractures

The fracture time was categorized by season as: spring (March to May), summer (June to August), autumn (September to November), and winter (December to February in the following year).

As shown in Table 1, there was no significant difference in the distribution of fracture types among different seasons (χ^2^ = 5.084, *P* = 0.166).

### Treatment and intraoperative blood transfusion

In all, 30 patients were treated non-surgically, and 1,302 patients received surgical treatment, accounting for 97.7% of the total cases. 864 patients with femoral neck fracture were treated with different surgical methods, of which total hip replacement was the major choice, making up 49.2%, followed by internal fixation, being about 24.4%. Among the patients with intertrochanteric fracture, 438 received surgical treatment, and the most common treatment was internal fixation, accounting for 97.8%. Among the 1,302 patients who underwent operation, 328 were given intraoperative blood transfusion, and 63.1% of them were patients with intertrochanteric fracture, and the rate was significantly higher than that in patients with femoral neck fracture.

### Trend of hospital stay and cost of hospitalization over a 6-year period

The median length of hospital stay lasted 8 days, and cost of hospitalization was 49,112.52 RMB. The hospital stay length dropped over the 6-year study period, whereas cost of treatment rose (*P* trend < 0.01) (Table 2).

**Table 2:** Trend analysis of hospital stay and cost of patients with hip fracture over a 6-year period

**Variable**	**Cases/persons-years**	**Hospital cost**			**Hospital stay**		
		**Median**	**OR (95%CI)**	***P* value**	**Median**	**OR (95%CI)**	***P* value**
year							
2015	190	48,160.72	1.0	-	9	1.0	-
2016	234	47,532.53	2,927.57 (-2,051.25, 7,906.39)	0.249	8	-0.40 (-1.56, 0.77)	0.505
2017	247	51,091.47	6,729.89 (1,810.13, 11,649.65)	0.007	8	-1.36 (-2.52, -0.21)	0.020
2018	294	48,005.28	4,578.38 (-167.32, 9,324.08)	0.059	8	-1.26 (-2.37, -0.15)	0.026
2019	253	52,879.29	6,485.74 (1,591.41, 11,380.07)	0.009	7	-2.28 (-3.42, -1.13)	0.000
2020	114	45,127.01	7,873.71 (1,833.72, 13,913.69)	0.011	7	-0.69 (-2.10, 0.73)	0.341
P for trend	1,332		1,336.44 (423.40, 2,249.49)	0.004		-0.35 (-0.56, -0.14)	0.001

*CI* Confidence Interval, *OR* odds ratio

Multivariate analysis was performed to detect factors that might be associated with hospital cost, and the result showed that intertrochanteric fracture (odds ratio (OR) = 12,021.93, 95% (confidence interval (CI) = 9591.13, 14452.73), treatments, perioperative blood transfusion (OR = 3,672.59, 95% CI = 1691.54, 5653.64), and length of hospital stay (OR = 2,023.11, 95% CI = 1888.44, 2157.77) were significantly associated with hospitalisation cost. After adjustments for age and gender, the hospitalisation cost remained statically significant (Table 3).

**Table 3:** Multivariable analysis of hospital cost for patients with hip fracture

**Variables**	**Model 1**		**Model 2**	
	**OR (95%CI)**	***P***	**OR (95%CI)**	***P***
Length of stay	2,023.11 (1,888.44, 2,157.77)	<0.001	2,029.80 (1,896.04, 2,163.56)	<0.001
Diagnosis				
Femoral neckfracture	0.00 (Reference)		0.00 (Reference)	
Intertrochanteric fracture	12,021.93 (9,591.13, 14,452.73)	<0.001	14,313.31 (16,934.82,11,691.82)	<0.001
Treatment				
non-surgical treatment	0.00 (Reference)		0.00 (Reference)	
Internal fixation	20,304.18 (14,888.80, 25,719.56)	<0.001	18,422.61 (12,966.92, 23,878.31)	<0.001
Hemiarthroplasty	41,179.33 (35,477.03, 46,881.63)	<0.001	42,601.19 (36,909.49, 48,292.90)	<0.001
Total hip replacement	58,588.31 (53,095.49,64,081.14)	<0.001	58,051.47 (52,586.12, 63,516.82)	<0.001
Perioperative blood				
No	0.00 (Reference)		0.00 (Reference)	
Yes	3,672.59 (1,691.54, 5,653.64)	<0.001	4,415.79 (2,425.47, 6,406.11)	<0.001

Model1, crude, Model2, adjusted for age, sex, *CI* Confidence Interval, *OR* odds ratio

## Discussion

Globally, the incidence of hip fractures is high, especially in the elderly with osteoporosis. Studies have shown that the overall incidence of hip fracture is still on the rise, especially in Asians [16, 17]. The incidence of hip fracture reportedly varies among countries and even within a country [18, 19]. The epidemiological investigation on hip fracture in different areas can inform and improve the prevention and treatment of hip fracture, and thereby mitigate the economic and medical burden on society and families.

The analysis of the onset age revealed that, with the increase of age, the number of female patients with femoral neck fracture and intertrochanteric fracture gradually exceeded that of male patients. This finding might be ascribed to the decreased osteoblast activity resulting from lowered estrogen level in postmenopausal women, and elevated parathyroid hormone, causing bone calcium loss. As a result, the incidence and severity of osteoporosis were higher in women than in men.

As age increased, the number of patients suffering from hip fractures in the 81–90 age group reached a peak. Among people younger than 90 years, the proportion of patients with femoral neck fractures were more than their counterparts with intertrochanteric fractures. In people older than 90 years, the patients with intertrochanteric fractures out-numbered those with femoral neck fractures. The results suggest that the elderly are more likely to suffer from intertrochanteric fractures. The possible reason is that, after an elderly person falls, the lateral trochanter of the hip joint tends to hit the ground. Due to the severe degree of osteoporosis, the impacting force cannot be transmitted, and the direct impact leads to the fracture of the trochanter. These characteristics indicate that major attention should be paid to the prevention of hip fracture in the elderly people, especially elderly females.

This study showed that hip fractures were more frequent in winter. Several studies have reported similar results [20, 21], although some studies exhibited that hip fractures were not seasonally related. The reason might be that the winter is cold and the clothes are thicker, which restricts body movement and makes them more likely to fall.

Our study also found that total hip arthroplasty (49.2%) was the most common treatment for femoral neck fractures, followed by internal fixation (24.4%). Among all types of treatment, internal fixation was the commonest (97.8%) for intertrochanteric fractures. Comparatively, the internal fixation is associated with less intraoperative blood loss, while the hip replacement takes a short time. Different surgical methods have their own advantages and disadvantages, so the appropriate operation method should be chosen according to the patient's situation.

Upon trend analysis, we found that the cost of hospitalization had significantly increased annually, whereas hospital stay time decreased. Decreased hospital stay length in recent years suggests improvements in medical technologies, nursing and rehabilitation care in our hospital, which might also partially explain the increase in the cost of hospitalization. The zoledronic acid (2009) and teriparatide (2012) were introduced to prevent osteoporosis and reduce the risk of hip [22], which, at the same time, increased the use of these drugs. In addition, the increase in the cost of surgery and related medical materials may also lead to an increase in the expense of each patient [23]. Population aging and rising costs in health care are likely to become two substantial challenges facing the next generation of clinicians and health policy makers. The cost of hospitalization could be influenced by various factors not adjusted for, including insurance, ward, nursing staff, and complications after the admission to the hospital. Further studies are needed to explore other underlying reasons.

Notably, the median hospitalization cost of femoral neck fractures was significantly higher than that of intertrochanteric fracture, which was also confirmed by multiple linear regression. This might be attributed to the fact that hip replacement was commonly applied for these patients, thus the cost of which was higher.

Therefore, proper medical management, social preventive measures, and prevention of falls are needed to reduce the risk of hip fracture and the socioeconomic burden. For example, improving health education for the elderly (especially for elderly women), early anti-osteoporosis treatment, and joint effort between medical staff and the community to build a safe environment for the elderly are all effective measures.

## Conclusion

This epidemiological study found that patients with hip fracture had certain distribution characteristics in age, sex, type of fracture, injury season, cause of injury, mode of operation, length of stay, treatment cost and so on. Proper medical management, social preventive measures, and prevention of falls are needed to reduce the risk of hip fracture and the socioeconomic burden.

## Limitations

This study has some limitations. First, the epidemiological and clinical characteristics of this study were confined to inpatients with hip fracture in a tertiary hospital in Shanghai. Second, other data regarding the factors associated with hospitalization cost were not available. Third, our dataset lacked detailed classification of surgical methods regarding “internal fixation” group and arthroplasty group.

## Data Availability

The datasets used and/or analyzed during the current study are available from the corresponding author on reasonable request.

## Abbreviations

SD: Standard Deviation
CI: confidence interval
OR: odds ratio
RMB: Ren Min Bi
